# A Fivefold Maximum Drug-Likeness Strategy for Prioritizing Antibacterial Candidates Against *Escherichia coli*

**DOI:** 10.3390/ph19050744

**Published:** 2026-05-08

**Authors:** Haoyu Zhu, Shijie Du, Qin Yang, Lu Xu, Wei Shi

**Affiliations:** 1 College of Material and Chemical Engineering, Tongren University, Tongren 554300, China; zhuhaoyu_98@163.com (H.Z.); dsj5216@163.com (S.D.); 2 School of Physics and Optoelectronic Engineering, Yangtze University, Jingzhou 434023, China; yangqin00055@hnu.edu.cn; 3 School of Sports and Health Science, Tongren University, Tongren 554300, China

**Keywords:** fivefold maximum drug-likeness, deep learning, virtual screening, antibacterial discovery, *Escherichia coli*, penicillin-binding protein 2

## Abstract

**Background/Objectives**: Early-stage antibacterial discovery requires balancing activity with broader developability-related properties. This study developed a Fivefold Maximum Drug-Likeness strategy (5F-MDL) as a multidimensional, developability-aware framework for prioritizing antibacterial candidates against *Escherichia coli* (*E. coli*). **Methods**: An ensemble of endpoint-specific deep learning models was used to construct a 33-dimensional predicted property spectrum. Approximately 16 million commercially available molecules were screened by comparing their normalized property profiles with those of clinically approved cephalosporin reference drugs. Fifteen top-ranked candidates were selected for experimental evaluation. Antibacterial activity was assessed by disk diffusion, minimum inhibitory concentration (MIC), and minimum bactericidal concentration (MBC) assays. Molecular docking, molecular dynamics simulations, MM-PBSA analysis, and a Bocillin-FL competition labeling assay were used as target-related supportive analyses. **Results**: Among the 15 prioritized candidates, three compounds showed measurable antibacterial activity against *E. coli* ATCC 25922. M2 showed the most favorable in vitro profile among the active candidates, with an MIC of 25.6 µg/mL and an MBC of 51.2 µg/mL, although its potency remained weaker than that of cefuroxime. Docking, molecular dynamics, and MM-PBSA analyses suggested that M2 could maintain a relatively stable noncovalent interaction pattern within the modeled PBP2 pocket, and the Bocillin-FL assay showed that M2 reduced fluorescent probe labeling of PBP2 in vitro. **Conclusions**: These findings provide preliminary proof-of-concept support for 5F-MDL as a multidimensional prioritization strategy for early-stage antibacterial candidate selection. M2 should be regarded as a preliminary antibacterial hit for further optimization rather than as a validated lead compound. Broader applicability, comparative advantage, and mechanistic relevance require further validation using larger candidate sets, resistant clinical isolates, systematic benchmarking, and direct target-validation experiments.

## 1. Introduction

Drug discovery remains a cornerstone of modern medicine and is essential for addressing diseases with substantial global burden, including cancer, human immunodeficiency virus/acquired immunodeficiency syndrome (HIV/AIDS), and tuberculosis [1,2,3,4,5,6]. Despite sustained advances in biomedical science, the discovery and development of new therapeutics remain inefficient, costly, and failure-prone. Only a small fraction of compounds that enter early discovery pipelines progress to clinical evaluation, and even fewer ultimately reach approval; the complete process commonly requires more than a decade [7,8,9]. This imbalance between urgent therapeutic demand and high developmental attrition has driven the search for more systematic, predictive, and resource-efficient drug discovery strategies.

The evolution of drug discovery has moved from empirical screening toward increasingly rational and computation-assisted design. Early trial-and-error approaches in activity screening and lead optimization provided the foundation for modern discovery practice [10,11,12,13]. Subsequently, high-throughput screening (HTS) and rational drug design (RDD) became widely adopted strategies. HTS allows rapid interrogation of large compound collections [14,15], but it is often limited by false-positive rates, high operational cost, technical complexity, and incomplete translation from primary hits to clinically useful candidates [16,17,18,19,20]. RDD improves molecular design by incorporating three-dimensional target structures and mechanistic knowledge [21,22,23], yet its effectiveness depends strongly on the quality of structural information and the reliability of target-mechanism assumptions [24,25,26]. Computer-aided drug design (CADD) has therefore become an important component of contemporary discovery pipelines. Common CADD strategies include molecular docking, quantitative structure–activity relationship (QSAR) modeling, molecular dynamics (MD) simulations, and pharmacophore modeling [27,28]. Docking is commonly used to estimate binding poses and relative affinities [29,30], QSAR models link molecular structure to measured or predicted activity [31,32], MD simulations provide insight into conformational stability and dynamic interactions [33,34], and pharmacophore models capture spatial arrangements of activity-related features for virtual screening [35,36,37]. These approaches have also contributed to rapid-response discovery efforts during public health emergencies [38,39]. Published antibacterial virtual screening campaigns have demonstrated that computer-aided prioritization can effectively narrow very large chemical spaces to experimentally testable subsets, but they also highlight a persistent translational bottleneck. In representative studies, virtual screening campaigns have ranged from approximately 1.5 million to nearly 6 million compounds, yet only a limited number of top-ranked molecules ultimately progressed to experimental testing or binding confirmation [40,41,42]. These outcomes illustrate a broader challenge in antibacterial discovery: favorable docking scores, activity-related predictions, or structural similarity alone do not necessarily translate into whole-cell antibacterial activity or broader developability. This gap is particularly important in Gram-negative discovery, where permeability, intracellular accumulation, efflux, solubility, and stability may all constrain experimental success even when target-oriented in silico ranking appears favorable.

Accordingly, a key unresolved problem is not merely how to identify molecules with favorable predicted affinity or activity, but how to prioritize molecules with a more balanced profile across multiple developability-related dimensions before experimental testing. Many current workflows remain centered on one dominant objective, such as docking rank, predicted activity, or compact drug-likeness descriptors. Although these approaches are valuable, they may not adequately capture the broader combination of physicochemical, pharmacokinetic, efficacy-related, safety-related, and stability-related factors that often determine whether a computationally attractive molecule can progress experimentally. Therefore, a systematic framework that centers on multidimensional developability, while balancing efficiency and translatability, is needed.

In response to these gaps, we propose Maximum Drug-Likeness (MDL) and formulate the Fivefold MDL (5F-MDL) paradigm. Here, “fivefold” refers not to five individual scalar parameters, but to five broader developability-related dimensions: physicochemical properties, pharmacokinetics, efficacy, safety, and stability. In this paradigm, the screening objective is integrated similarity to approved drugs across these five dimensions. To realize this objective, we used large-scale molecular structure and activity data to build, within an ensemble deep learning framework, a set of QSAR models for predicting properties across the five dimensions. These models collectively generate a 33-dimensional property spectrum intended to represent multidimensional developability-related behavior rather than a minimal descriptor set. Candidate molecules were then prioritized according to their integrated similarity to approved drugs across the five dimensions. We further used experimental validation together with molecular docking and molecular dynamics analyses to examine whether prioritized compounds show activity and target-relevant interaction patterns consistent with the screening predictions [27,28,29,30,31,32,33,34]. Thus, the intended role of 5F-MDL is not to replace conventional docking, QSAR, or ligand-based screening, but to function as a multidimensional, developability-aware prioritization framework upstream of experimental testing.

Within this framework, we adopted *Escherichia coli* (*E. coli*) as the reference pathogen for molecular selection and evaluated the 5F-MDL method in a Gram-negative antibacterial setting. *E. coli* is a common Gram-negative opportunistic pathogen and a major public health concern [43,44,45,46]. In 2024, the World Health Organization (WHO) continued to classify carbapenem-resistant Enterobacteriaceae (CRE) as critical priority pathogens [47]. *E. coli* is a leading cause of urinary tract infections and is associated with multiple hospital-acquired infections [48,49]. Clinical treatment has relied primarily on β-lactam antibiotics that target penicillin-binding proteins (PBPs), with penicillin-binding protein 2 (PBP2) playing a pivotal role [50,51,52]. However, enzyme-mediated hydrolysis, reduced membrane permeability, target-site mutations, and bypass pathways limit drug affinity and exposure, leading to therapeutic failure [53,54,55,56,57,58,59,60,61,62,63]. The global burden of resistance continues to rise, while innovation in new scaffolds for Gram-negative pathogens remains limited [64,65,66,67,68]. Against this backdrop, the 5F-MDL paradigm was designed to prioritize molecules with balanced predicted profiles across physicochemical, pharmacokinetic, efficacy-related, safety-related, and stability-related dimensions while retaining relevance to antibacterial target engagement such as PBP2-related activity [50,51,52,56,57,58,59].

In summary, we developed a 5F-MDL strategy and an ensemble deep learning-based screening workflow for *E. coli*-related molecular prioritization. By integrating multidimensional property prediction, virtual screening, molecular docking, molecular dynamics analysis, and experimental evaluation, this study was designed to explore whether a developability-aware prioritization framework could support early-stage antibacterial candidate selection while reducing reliance on single-dimension optimization. At the same time, the present work should be interpreted as an initial methodological and feasibility-oriented study rather than as definitive evidence of broad general applicability or superiority over existing prioritization approaches.

## 2. Results

### 2.1. Performance Evaluation of Predictor Submodels

To evaluate whether the predicted properties could provide a quantitative basis for constructing the multidimensional property spectrum, the 33 endpoint-specific deep learning submodels were assessed using endpoint-wise held-out test sets. The predictive performance of these submodels is summarized in Table 1, and the composition of the corresponding property datasets and submodels is provided in Table S1. These evaluations were intended to assess the internal predictive reliability of the individual property models within the current workflow, rather than to serve as definitive evidence of broad external generalizability.

Overall, favorable predictive performance was observed across both regression and classification tasks. For the 27 regression models (S1–S27), the coefficient of determination ($R^{2}$) ranged from 0.86 to 0.96, with a mean $R^{2}$ of approximately 0.90. Models targeting physicochemical properties (S1–S11) showed consistently strong performance, with a mean $R^{2}$ exceeding 0.91. Several pharmacokinetic-related endpoints that are generally challenging to model, including bioavailability (S12, $R^{2}$ = 0.88) and clearance (S16, $R^{2}$ = 0.88), also showed acceptable predictive performance under the present modeling conditions. These results suggest that the deep learning submodels captured useful structure–property relationships for the endpoint-specific datasets used in this study.

For the six classification models (S28–S33) related to safety and stability, the mean area under the receiver operating characteristic curve (ROC AUC) was approximately 0.94. In particular, the Ames mutagenicity model (S29) and the human ether-à-go-go-related gene (hERG) toxicity risk model (S30) achieved AUC values of 0.96 and 0.95, respectively. These results support the use of the 33 predicted endpoints as a practical property spectrum for subsequent 5F-MDL-based prioritization. However, because the current evaluation was based on held-out test sets rather than a fully independent external or scaffold-split validation scheme, the possibility of residual structural relatedness or endpoint-specific overfitting cannot be completely excluded and is considered further in the Discussion.

### 2.2. Analysis of Property Spectra for Candidate Drugs

**Figure 1:**
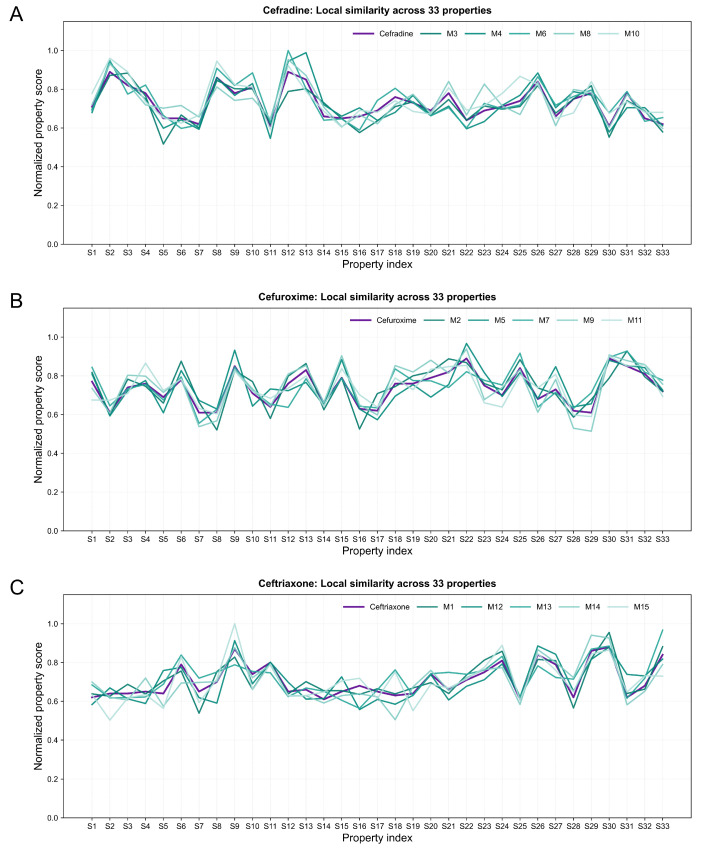
Comparison of multidimensional property profiles between reference drugs and representative candidate molecules in the 33-dimensional property space. Normalized predicted values are shown for (**A**) cefradine, (**B**) cefuroxime, and (**C**) ceftriaxone, together with their respective top five candidate molecules. In each panel, the reference drug is shown as a thick purple line, whereas the top five candidate molecules are shown as thin teal-gradient lines. The overall similarity of the curve patterns reflects similarity in the predicted multidimensional property profiles between candidate molecules and their corresponding reference drugs.

Based on the trained submodels, 33-dimensional predicted property spectra were generated for the three reference drugs (cefradine, cefuroxime, and ceftriaxone) and for the prioritized candidate molecules. For each reference drug, candidate molecules with the smallest Euclidean distances in the normalized 33-dimensional space were selected and visualized together as property fingerprint spectra (Figure 1), allowing qualitative comparison of the multidimensional predicted profiles between reference and candidate molecules.

In these spectra, the x-axis represents the 33 predicted properties, and the y-axis represents the normalized property values ranging from 0 to 1. Each reference drug shows a characteristic multidimensional profile across physicochemical, pharmacokinetic, efficacy-related, safety-related, and stability-related endpoints. The corresponding candidate molecules display generally similar trajectories, indicating that the prioritized molecules preserved broad profile-level similarity to their associated reference drugs in the normalized property space.

Among the phenotypically active candidates, M8 was grouped with the cefradine-associated candidates, whereas M2 and M9 were grouped with the cefuroxime-associated candidates in Figure 1. In all three reference-centered panels, the top-ranked candidates broadly followed the overall peak–trough patterns of the corresponding reference drugs, suggesting that the Euclidean-distance-based selection procedure was able to enrich molecules with similar predicted multidimensional property profiles. This visual concordance provides profile-level support for the 5F-MDL prioritization strategy, although it should be interpreted as evidence of predicted property similarity rather than as direct proof of equivalent antibacterial activity or developability.

### 2.3. Virtual Screening Results and $S_{5F}$ Prioritization

The commercial screening libraries from eMolecules, TargetMol, and MedChemExpress (MCE), comprising approximately 16 million commercially available molecules in total, were ranked according to the 5F-MDL prioritization workflow. For each molecule, a 33-dimensional predicted property spectrum was generated and compared with the corresponding reference drug profile. A composite similarity score, $S_{5F}$, was then calculated based on Euclidean distance in the normalized 33-dimensional property space. For each reference drug, the five molecules with the highest similarity scores were retained, yielding 15 prioritized candidates for subsequent evaluation (Table 2).

All 15 prioritized molecules showed $S_{5F}$ similarity scores above 0.93, and the top-ranked molecule within each reference-centered set exceeded 0.955, indicating close predicted proximity to the corresponding reference drug in the multidimensional property space (Table 2). Among the phenotypically active candidates identified later in the study, M2 and M9 were prioritized in relation to cefuroxime, whereas M8 was prioritized in relation to cefradine. For example, M2 showed high similarity to cefuroxime in the integrated property space and also exhibited a lower predicted hERG toxicity risk than the cefuroxime reference for endpoint S30 (0.55 vs. 0.61), representing a favorable predicted difference in this safety-related dimension (Table 2). These results indicate that the $S_{5F}$ procedure selected molecules with high predicted multidimensional similarity to the selected reference drugs, although such similarity should be interpreted as a prioritization criterion rather than as direct evidence of antibacterial activity.

### 2.4. Antibacterial Activity by Disk Diffusion

**Figure 2:**
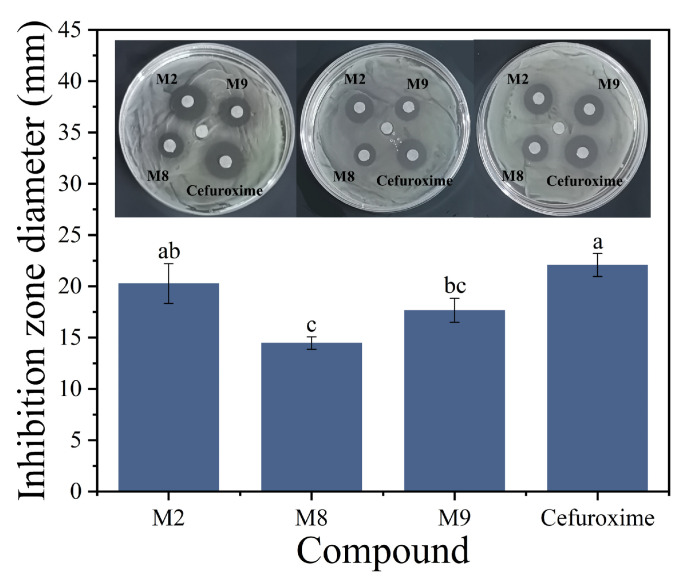
In vitro antibacterial activity of prioritized candidate molecules against *Escherichia coli* ATCC 25922. Representative inhibition zones from three independent biological replicates are shown for M2, M8, and M9 in comparison with cefuroxime at a loading of 30 µg per disk. Bar plots show inhibition zone diameters as mean ± SD (*n* = 3 independent biological replicates). Statistical significance among treatments was analyzed by one-way analysis of variance (ANOVA) followed by Tukey’s multiple-comparison test. Different letters above the bars indicate statistically significant differences among groups (*p* < 0.05).

Phenotypic antibacterial activity of the 15 prioritized candidates was evaluated using a modified disk diffusion assay against *E*. *coli* ATCC 25922. Under the present assay conditions, most candidates produced no discernible inhibition zones at a loading of 30 µg per disk. In contrast, only three compounds, namely M2, M8, and M9, generated clear zones of inhibition (Figure 2), indicating measurable antibacterial activity in this primary phenotypic screen.

Among the active compounds, M2 produced the largest inhibition zone diameter (20.27 ± 1.94 mm), which was numerically close to that of the positive control cefuroxime (22.07 ± 1.12 mm). M9 showed intermediate activity (17.66 ± 1.17 mm), whereas M8 produced the smallest inhibition zone among the three active candidates (14.47 ± 0.60 mm). Statistical analysis showed that the inhibition zones differed significantly among treatments under the present experimental conditions (Figure 2). Based on these results, M2, M8, and M9 were selected for subsequent determination of the minimum inhibitory concentration (MIC) and minimum bactericidal concentration (MBC).

### 2.5. MIC and MBC Determination Results

Antibacterial potency was further quantified by broth microdilution following the CLSI M07-A9 guideline. Against *E. coli* ATCC 25922, the MIC values were 25.6 µg/mL for M2 and M8 and 51.2 µg/mL for M9, whereas the reference antibiotic cefuroxime showed a lower MIC of 3.2 µg/mL (Table 3). These results indicate that M2, M8, and M9 exhibited measurable antibacterial activity under the tested conditions, but their potencies remained substantially lower than that of cefuroxime.

Bactericidal activity was evaluated by MBC determination. M2 showed an MBC of 51.2 µg/mL, whereas M8 and M9 both had MBC values of 102.4 µg/mL (Table 3). The MBC/MIC ratios were 2 for M2, 4 for M8, and 2 for M9, meeting the conventional criterion (≤4) for bactericidal activity under the present experimental conditions. Considering MIC, MBC, and MBC/MIC together, M2 showed the most favorable in vitro profile among the three active candidates. Nevertheless, given its weaker potency relative to cefuroxime, M2 is more appropriately regarded as a preliminary antibacterial hit for further optimization rather than as a fully validated lead compound.

### 2.6. Molecular Docking Results

Molecular docking was performed for the 15 prioritized candidates and the three reference antibiotics to evaluate their potential noncovalent interactions with the modeled PBP2 binding pocket. For most molecules, the most favorable docking poses were obtained in Cavity 1 (estimated volume ≈ 517 Å^3^), indicating a generally consistent pocket preference across ligands (Table S2). Overall, the prioritized candidates achieved a mean docking score of −9.9 kcal/mol, whereas the three reference drugs showed a mean docking score of −8.4 kcal/mol (Table S2). However, docking rank did not uniformly correspond to phenotypic antibacterial activity, indicating that docking score alone was insufficient to explain the biological performance of this compound set.

To facilitate comparison with the biological results, the docking outcomes of the three phenotypically active candidates are summarized in Table 4. Among these compounds, M8 gave the most favorable docking score (−11.3 kcal/mol), followed by M9 (−10.5 kcal/mol) and M2 (−9.8 kcal/mol), whereas the reference drug cefuroxime showed a docking score of −8.5 kcal/mol. Notably, the best docking score did not correspond to the strongest antibacterial activity, again suggesting that noncovalent docking alone could not fully account for whole-cell activity under the tested conditions.

**Figure 3:**
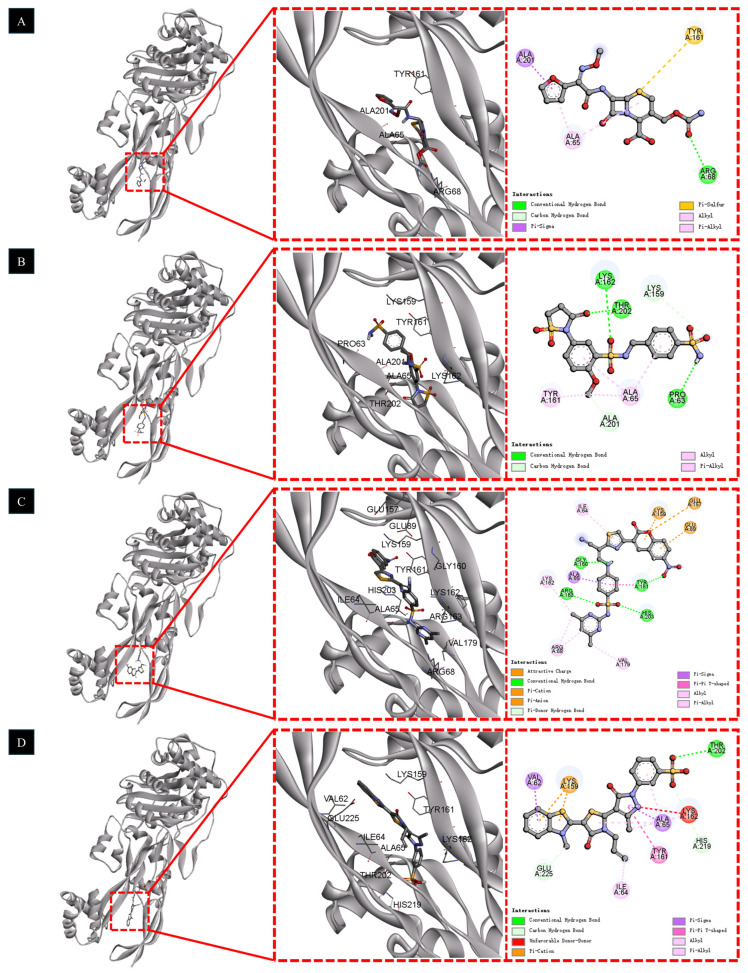
Predicted binding modes of cefuroxime and the three phenotypically active candidate molecules within the modeled active pocket of PBP2. Docking poses are shown for (**A**) cefuroxime, (**B**) M2, (**C**) M8, and (**D**) M9, illustrating their predicted binding orientations and representative interactions within the catalytic cavity.

Structural inspection of the docking poses was therefore used as a supportive analysis to compare how cefuroxime, M2, M8, and M9 were positioned within the PBP2 active pocket and to identify plausible interaction features associated with their different experimental behaviors (Figure 3). As the positive control with the strongest in vitro antibacterial activity, cefuroxime was docked in a pre-reaction (pre-acylation) state. In the predicted pose, the β-lactam-containing scaffold was positioned in the active groove and was associated with a π-alkyl interaction involving ALA65, together with a conventional hydrogen bond between ARG68 and the carbamate side chain. Although its noncovalent docking score (−8.5 kcal/mol) was not the most favorable in the set, this pose may still be compatible with productive positioning prior to covalent inhibition. This interpretation should, however, be regarded as a docking-based structural hypothesis rather than as definitive mechanistic evidence.

Among the three active candidates, M2, which showed the most favorable overall antibacterial profile in vitro, displayed a binding mode characterized by multiple predicted polar contacts. As shown in Figure 3B, conventional hydrogen bonds were predicted between the sultam/sulfonamide region of M2 and residues THR202 and LYS162. In addition, carbon-hydrogen bond interactions involving LYS159 and ALA201, together with a hydrophobic contact at ALA65, were also observed in the predicted pose. These interactions provide a plausible structural rationale for the experimentally observed activity of M2, although they do not by themselves establish PBP2 inhibition as the primary mechanism of action.

Although M8 yielded the most favorable docking score (−11.3 kcal/mol), its in vitro antibacterial performance was weaker than that of M2. The predicted binding mode of M8 involved a combination of polar and hydrophobic contacts, including interactions with GLU89, GLU157, LYS159, VAL179, and ARG68. These interactions may have contributed to the favorable docking score, but they did not translate into the strongest phenotypic activity under the tested conditions. This discrepancy further supports the view that docking score alone is not sufficient to predict antibacterial performance and that other factors, such as solubility, permeability, intracellular accumulation, or other developability-related constraints, may also play important roles.

M9 showed a distinct predicted binding mode. Its sulfonic acid group formed hydrogen bonds with THR202, indicating favorable polar engagement with the pocket. At the same time, the docking model suggested an unfavorable donor-donor contact between a nitrogen atom in the core scaffold and LYS162 (Figure 3D, red dashed line). This feature may impose a local energetic penalty and may help explain why M9, despite occupying the pocket, showed weaker antibacterial potency than M2. Nevertheless, this interpretation remains tentative and should be viewed as a hypothesis that may guide future scaffold optimization rather than as a definitive causal explanation.

### 2.7. Molecular Dynamics Simulation

To further examine the stability of the modeled protein–ligand complexes, molecular dynamics simulations were performed for the M2–PBP2 complex and the cefuroxime–PBP2 reference complex. The purpose of these simulations was to evaluate the conformational stability and interaction patterns of representative bound complexes at the atomic level, rather than to directly simulate concentration-dependent competition or to provide definitive mechanistic validation. Root-mean-square deviation (RMSD), root-mean-square fluctuation (RMSF), trajectory-based interaction occupancies, and molecular mechanics Poisson–Boltzmann surface area (MM-PBSA) energy components were analyzed.

**Figure 4:**
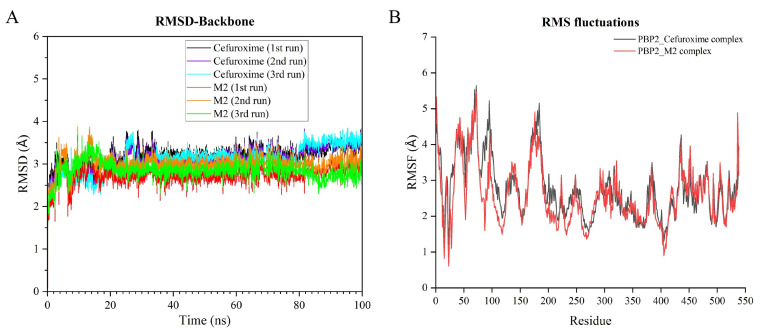
Stability analysis from molecular dynamics simulations of complexes formed by cefuroxime or candidate M2 with PBP2. (**A**) Time trajectories of root-mean-square deviation (RMSD) values for the complexes. (**B**) Root-mean-square fluctuation (RMSF) profiles of protein residues. Values are reported in Å.

Overall structural stability and residue-level flexibility were first assessed using RMSD and RMSF, respectively. The initial structures were used as references, and the time evolution of RMSD for both systems is shown in Figure 4A. Within the first 20 ns, the trajectories reached relatively stable fluctuation ranges, indicating that the simulated complexes remained stable under the applied conditions. After equilibration, the M2–PBP2 complex exhibited a mean RMSD of 2.8 ± 0.2 Å, whereas the cefuroxime–PBP2 complex showed a mean RMSD of 3.3 ± 0.3 Å. This difference suggests that, under the present simulation conditions, the modeled M2–PBP2 complex maintained a relatively stable noncovalent binding state. However, this result should be interpreted as evidence of simulation-level conformational stability rather than as direct proof of antibacterial mechanism.

Subsequent RMSF analysis (Figure 4B) showed generally similar residue-fluctuation profiles for the two simulated systems, with local differences in regions surrounding the modeled binding pocket. In particular, residues 50–100 showed higher flexibility in the cefuroxime–PBP2 system, whereas the M2–PBP2 system showed reduced fluctuation in this region. A similar trend was observed around residues 190–210, which includes residues close to the modeled active-site region. These observations suggest that M2 may stabilize local regions around the modeled pocket under the simulation conditions. Because RMSF alone cannot identify the specific interaction responsible for these local effects, trajectory-based interaction occupancies were further analyzed.

To provide a more quantitative description of noncovalent interactions, the mean number of hydrogen bonds and the occupancies of major hydrogen-bonding, hydrophobic, and π–π interactions were calculated from the molecular dynamics trajectories (Table 5). The M2–PBP2 complex showed a higher mean number of hydrogen bonds (2.634) than the cefuroxime–PBP2 complex (1.773). In the M2–PBP2 system, high-occupancy hydrogen bonds were observed with LYS162 (97.9%) and THR202 (98.6%), whereas the cefuroxime–PBP2 system showed high-occupancy hydrogen bonding mainly with ARG68 (99.3%) and PRO66 (78.0%). Major hydrophobic contacts were also observed in both systems, including ALA65 and TYR161, while π–π interactions involving TYR161 occurred with relatively low occupancy in cefuroxime–PBP2 (3.6%) and higher, although still moderate, occupancy in M2–PBP2 (12.7%). These trajectory-based results support the presence of persistent noncovalent interaction networks in the modeled M2–PBP2 complex, but they should still be interpreted as computational support rather than conclusive target validation.

To further quantify interaction energetics, binding free energies for the final 10 ns of each trajectory were calculated using the MM-PBSA method (Table 6). The M2–PBP2 complex showed a binding free energy of −38.54 kcal/mol, which was more favorable than that of the cefuroxime–PBP2 complex (−29.40 kcal/mol). Energy decomposition indicated that the M2–PBP2 interaction was mainly supported by van der Waals (−45.21 kcal/mol) and electrostatic (−28.65 kcal/mol) contributions, partly offset by an unfavorable polar solvation term (+39.12 kcal/mol) and a favorable nonpolar solvation term (−3.80 kcal/mol). For cefuroxime–PBP2, the corresponding van der Waals, electrostatic, polar solvation, and nonpolar solvation contributions were −35.40, −18.22, +27.08, and −2.86 kcal/mol, respectively. In the present analysis, the polar and nonpolar solvation terms were used to describe solvation-related contributions associated with desolvation effects.

Taken together, the RMSD/RMSF profiles, interaction occupancies, and MM-PBSA energy components suggest that M2 can maintain a relatively stable noncovalent interaction pattern within the modeled PBP2 pocket under the computational conditions used in this study. Nevertheless, these results represent supportive structural and energetic evidence only. They do not establish PBP2 as the primary intracellular target of M2 or prove the antibacterial mechanism of action.

### 2.8. Bocillin-FL Competition Assay

A competitive labeling assay based on the fluorescent β-lactam probe Bocillin-FL was performed to further examine whether M2 could interfere with probe labeling of PBP2 in vitro. As shown in Figure S2A, a distinct fluorescent band corresponding to PBP2 was observed in the control group treated with Bocillin-FL alone, indicating effective probe labeling of the protein under the assay conditions. When M2 was added at increasing concentrations from 25 to 100 µM, the fluorescence intensity of the PBP2 band gradually decreased. In comparison, the positive control cefuroxime almost completely suppressed the Bocillin-FL labeling signal. These results indicate that M2 can reduce Bocillin-FL labeling of PBP2 in a concentration-dependent manner under the present in vitro conditions.

Densitometric analysis of the fluorescent bands (Figure S2B) further supported this trend, showing a gradual reduction in relative PBP2 labeling intensity with increasing M2 concentration. Because Bocillin-FL forms an irreversible covalent label with PBPs, whereas M2 was modeled as a noncovalent ligand in the present computational workflow, this assay should be interpreted as evidence that M2 can interfere with probe labeling at or near the PBP2 active-site region rather than as definitive proof of direct enzymatic inhibition. In addition, the concentration-dependent Bocillin-FL assay and the molecular dynamics simulations address different experimental and computational levels: the former evaluates dose-dependent biochemical competition in vitro, whereas the latter examines the stability and interaction pattern of a representative bound complex at the atomic level. Therefore, the present Bocillin-FL results provide target-related supportive evidence for interaction between M2 and PBP2, but they do not establish PBP2 as the primary intracellular target or fully define the antibacterial mechanism of M2.

## 3. Discussion

This study explored a multidimensional prioritization strategy for early-stage antibacterial discovery by integrating 33 predicted properties across five developability-related dimensions: physicochemical properties, pharmacokinetics, efficacy-related endpoints, safety, and stability. The purpose of this framework was not to replace conventional docking, classical QSAR, fingerprint-based similarity screening, or other virtual screening approaches. Rather, 5F-MDL was designed as a developability-aware prioritization layer intended to help rank molecules before experimental testing by considering a broader property spectrum than activity or affinity alone. The favorable performance of the 33 endpoint-specific submodels on held-out test sets supports their use within the current workflow, but these metrics should not be interpreted as definitive evidence of broad external generalizability or superiority over established prioritization methods.

An important limitation of the current study is that it did not include a formal head-to-head benchmark against conventional docking-only ranking, classical QSAR, QED-based scoring, fingerprint similarity, or simplified multiparametric scoring strategies. Therefore, the present results cannot establish that 5F-MDL is categorically superior to these established approaches. The current validation of the 33 submodels was also based on held-out test sets rather than fully independent scaffold-split or external validation sets. Although the downstream screening libraries were not intentionally incorporated as complete training sources, limited structural overlap or close chemical relatedness between public training data and commercial screening libraries cannot be completely excluded. These issues should be addressed in future work through scaffold-aware validation, external benchmarking, and prospective testing across structurally diverse chemical series.

The use of a 33-dimensional property space also requires careful interpretation. Compact drug-likeness frameworks such as QED are valuable because they summarize key oral drug-likeness descriptors, including molecular weight, lipophilicity, hydrogen-bonding capacity, polar surface area, rotatable bonds, aromatic rings, and structural alerts. In contrast, the present 33-dimensional space was intended to function as an endpoint-oriented property spectrum spanning multiple developability-related domains rather than as a minimal descriptor index. This broader coverage may be useful for early-stage prioritization, but it also increases model complexity and may reduce interpretability. Therefore, future studies should compare the 33-dimensional formulation with QED-based, PCA-reduced, or rationally selected smaller descriptor sets to determine whether similar or improved prioritization performance can be achieved with fewer variables.

A central outcome of this study is that only a subset of the 15 prioritized molecules showed measurable whole-cell antibacterial activity. Specifically, M2, M8, and M9 produced detectable inhibition under the tested conditions, whereas the remaining candidates did not show the desired phenotypic response. This attrition should be viewed as an important limitation of the current framework rather than as a negligible deviation. It indicates that high predicted multidimensional similarity to approved antibiotics does not guarantee whole-cell antibacterial activity. In Gram-negative bacteria, this gap is particularly plausible because antibacterial performance depends not only on target-related compatibility, but also on outer-membrane permeability, intracellular accumulation, active efflux, aqueous solubility, compound stability, and assay-relevant exposure. These factors are not fully captured by docking score alone and may also not be sufficiently represented in the current weighting of the 5F-MDL property spectrum.

The comparison among cefuroxime, M2, M8, and M9 further illustrates this point. M2 was prioritized in relation to cefuroxime and showed the most balanced in vitro profile among the active candidates. However, its MIC remained substantially higher than that of cefuroxime, indicating that M2 should be regarded as a preliminary antibacterial hit for further optimization rather than as a fully validated lead compound. Table 4 shows that M8 had the most favorable docking score among the three active candidates, yet its phenotypic activity was weaker than that of M2. This discrepancy indicates that docking-based ranking alone was insufficient to explain the observed antibacterial performance. In addition, the SwissADME-based comparison in Table S3 suggested that M2 had a more balanced developability-related profile than M8 and M9, particularly with respect to solubility-related behavior, lipophilicity, medicinal-chemistry alerts, and lead-likeness violations. These observations provide a plausible explanation for why M2 translated more favorably in the biological assays, although they do not prove that these descriptors were the sole determinants of activity.

These findings also suggest a practical direction for improving the 5F-MDL framework. The current model appears to enrich molecules with favorable multidimensional similarity to reference antibiotics, but it does not yet sufficiently filter out compounds that fail at the whole-cell level. Future versions of the framework may benefit from retraining, reweighting, or extending the property spectrum with endpoints more directly related to Gram-negative antibacterial performance, including outer-membrane permeability, intracellular accumulation, efflux susceptibility, aggregation tendency, and assay-relevant solubility. For Gram-negative antibacterial discovery, such exposure-related features may need to receive greater weight than they would in a general drug-likeness framework.

Another limitation concerns the similarity-based nature of the 5F-MDL strategy. Because the method is anchored to the multidimensional profiles of approved antibiotics, it may preferentially select molecules that remain close to known antibacterial developability space. This design is useful for early-stage prioritization because clinically used antibiotics provide realistic reference anchors. However, it may also introduce bias toward molecules analogous to known scaffolds and may limit the ability to identify entirely novel antibacterial chemotypes. Therefore, the current implementation should be viewed as a similarity-guided, developability-aware prioritization strategy with some potential for scaffold hopping, rather than as an unbiased engine for discovering completely unprecedented antibacterial chemical matter.

The structural and energetic analyses provide useful but limited support for target-related interaction. Docking suggested that M2, M8, and M9 can occupy the modeled PBP2 pocket, while molecular dynamics simulations further suggested that the M2–PBP2 complex can maintain a relatively stable noncovalent interaction pattern under the computational conditions used here. The trajectory-based interaction analysis in Table 5 showed that M2 maintained high-occupancy hydrogen bonds with LYS162 and THR202, and the MM-PBSA decomposition in Table 6 indicated favorable van der Waals and electrostatic contributions that were partially offset by polar solvation. These data strengthen the quantitative description of the modeled interaction, but they remain computational support. Docking, RMSD/RMSF analysis, interaction occupancies, and MM-PBSA calculations should be interpreted as hypothesis-generating evidence, not as definitive validation of the antibacterial mechanism.

The Bocillin-FL competition assay provided additional target-related biochemical support by showing that M2 reduced fluorescent probe labeling of PBP2 in vitro. Nevertheless, this assay does not by itself prove direct functional inhibition of PBP2 or establish PBP2 as the primary intracellular target of M2. Bocillin-FL forms an irreversible covalent label with PBPs, whereas M2 was modeled as a noncovalent ligand in the present computational workflow. Thus, the assay is best interpreted as evidence that M2 can interfere with probe labeling at or near the PBP2 active-site region. The concentration-dependent Bocillin-FL experiment and the molecular dynamics simulations also address different levels of analysis: the former reflects dose-dependent biochemical competition, whereas the latter evaluates the stability of representative bound complexes at the atomic level. Direct functional enzyme-inhibition assays, resistant mutant profiling, genetic perturbation, or other orthogonal target-validation approaches will be required to clarify the mechanism more rigorously.

The experimental validation also has limited translational scope. All antibacterial testing in the present study was performed using the susceptible reference strain *E. coli* ATCC 25922. This design is appropriate for an initial phenotypic evaluation of prioritized candidates, but it does not establish activity against resistant clinical isolates or clinically heterogeneous Gram-negative strains. Because the manuscript is positioned within the broader context of antimicrobial resistance, future work should test optimized candidates against resistant *E. coli* isolates and other clinically relevant Gram-negative strains to better assess translational potential.

Finally, the experimental validation scale remains limited. Testing 15 prioritized compounds from a screening space of approximately 16 million molecules provides preliminary prospective evidence of feasibility, but it is not sufficient to establish the overall predictive utility, generalizability, or comparative advantage of the framework. Larger prospective validation sets, structurally diverse candidate pools, and systematic comparisons with established prioritization baselines will be necessary to determine whether 5F-MDL can reliably improve antibacterial candidate selection.

In summary, the present study provides preliminary proof-of-concept support for a multidimensional, developability-aware prioritization workflow in antibacterial discovery. The identification of M2 as the most balanced candidate among the tested compounds suggests that the 5F-MDL strategy can help enrich experimentally testable molecules from large screening libraries. However, the high attrition rate, modest potency of M2 relative to cefuroxime, limited validation scale, lack of resistant-strain testing, absence of formal benchmarking, and incomplete mechanistic validation all indicate that the current framework remains at an early stage of development. Future work should focus on larger-scale prospective validation, systematic benchmarking, Gram-negative-specific model refinement, and more direct target-validation experiments.

## 4. Materials and Methods

### 4.1. MDL and 5F-MDL

Maximum Drug-Likeness (MDL) was defined in this study as a prioritization principle that seeks candidate molecules with high integrated similarity to approved reference drugs across multiple developability-related properties. Based on this principle, we formulated the Fivefold Maximum Drug-Likeness (5F-MDL) strategy. Here, “fivefold” refers to five broad developability-related dimensions, rather than five individual parameters: physicochemical properties, pharmacokinetics, efficacy-related properties, safety, and stability. Each dimension was represented by multiple endpoint-specific deep learning quantitative structure–activity relationship (QSAR) submodels. Under the 5F-MDL framework, candidate molecules were prioritized according to their integrated similarity to approved reference drugs across the five dimensions. The computational workflow was implemented using the commercial 5FMDL Screener platform (Real-Drug Technology Co., Ltd., Shanghai, China; www.5fmdl-screener.top).

The 5F-MDL framework was designed as a multidimensional property-spectrum-based workflow rather than as a compact descriptor index. In total, 33 endpoint-specific properties were selected according to the availability of curated QSAR-compatible datasets and their relevance to the five developability-related dimensions. These 33 endpoints were used to construct a predicted property spectrum for each molecule. The composition of the 33 property datasets and the corresponding predictive submodels is summarized in Table S1.

The physicochemical dimension describes molecular attributes that influence solubility, permeability, and formulation feasibility. Representative properties in this dimension include molecular weight, calculated partition coefficient (cLogP), distribution coefficient (logD), acid dissociation constant (pKa), hydrogen-bond donor and acceptor counts, topological polar surface area (tPSA), and aqueous solubility. These properties were included because approved drugs generally occupy characteristic physicochemical ranges that support practical developability.

The pharmacokinetic dimension describes the absorption, distribution, metabolism, and excretion (ADME) behavior of candidate molecules. It includes properties related to absorption and bioavailability, distribution behavior such as volume of distribution and protein binding, metabolic transformation and clearance, and terminal half-life. These properties were included to represent systemic exposure and disposition-related behavior.

The efficacy-related dimension refers to predicted pharmacological or bioactivity-related endpoints derived from available experimental data. These include potency-related metrics such as half-maximal inhibitory concentration (IC50), half-maximal effective concentration (EC50), and minimum inhibitory concentration (MIC), when such data were available for model construction. This dimension was intended to provide activity-related information within the broader multidimensional property spectrum, rather than to replace direct biological testing.

The safety dimension describes predicted tolerability and risk-related properties. It includes endpoints related to cytotoxicity, organ toxicity, genotoxicity, cardiovascular liability such as inhibition of the human ether-à-go-go-related gene (hERG) channel, hepatic and renal risks, immunological or endocrine effects, drug–drug interaction liability, and therapeutic-index-related considerations. The purpose of this dimension was to reduce prioritization of molecules with obviously unfavorable predicted safety profiles.

The stability dimension describes the ability of a molecule to preserve its chemical structure and functional properties during research, formulation, storage, and use. It includes chemical and physical stability, such as thermal, photolytic, oxidative, and hydrolytic stability, as well as solid-state stability, solution stability, plasma stability, metabolic stability, and compatibility with storage or transport requirements. These properties were included because molecular instability can limit the practical value of otherwise promising candidates.

The resulting 33-dimensional property spectrum was therefore intended to provide a broad developability-aware representation of each molecule. It should not be interpreted as the only possible descriptor set or as a proven optimal reduced feature space. Rather, it represents the current operational implementation of the 5F-MDL framework based on available endpoint datasets and the intended five-dimensional developability coverage.

### 4.2. Data

#### 4.2.1. Clinically Approved Reference Antibiotic Set

**Figure 5:**
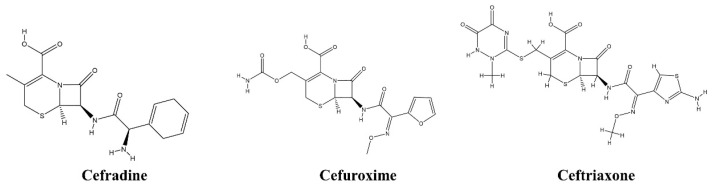
Chemical structures of the three clinically approved cephalosporin antibiotics used as reference drugs to define the 5F-MDL similarity space. Cefradine, cefuroxime, and ceftriaxone were selected as reference compounds for multidimensional drug-likeness calibration and similarity-based prioritization.

Three clinically approved cephalosporin antibiotics with documented antibacterial activity against susceptible *E*. *coli* strains were selected as reference drugs for defining the 5F-MDL similarity space. These agents belong to the β-lactam class and exert antibacterial activity through interaction with penicillin-binding proteins (PBPs), which are essential enzymes involved in bacterial cell wall biosynthesis. In the present study, PBP2 was selected as the target-relevant structural reference for docking and molecular dynamics analyses. Therefore, these reference antibiotics were used primarily as clinically established anchors for multidimensional property-spectrum comparison and PBP-related structural evaluation, rather than as evidence that all prioritized candidates necessarily share an identical mechanism of action. The selected reference drugs are clinically used for infections caused by susceptible bacteria, including susceptible *E. coli* infections in appropriate therapeutic contexts. Their inclusion was intended to provide realistic reference profiles for antibacterial drug-likeness and developability-related comparison. The names and chemical structures of the three reference antibiotics are shown in Figure 5.

#### 4.2.2. Candidate Molecule Set

The virtual screening libraries were compiled from commercially available compound collections, including eMolecules (San Diego, CA, USA), TargetMol (Wellesley Hills, MA, USA), and MedChemExpress (Shanghai, China). Together, these libraries provided a screening space of approximately 16 million commercially available small molecules with diverse chemical structures. These compound collections were used as the input chemical space for 5F-MDL-based computational prioritization. After ranking against the corresponding reference-drug profiles, the top five candidates associated with each reference drug were selected, resulting in 15 prioritized candidate molecules. These 15 top-ranked molecules were then purchased and subjected to subsequent experimental evaluation. Because the screening libraries represent broad commercial chemical space and the endpoint-specific QSAR datasets were derived from publicly available molecular data sources, limited structural overlap or close chemical relatedness between training data and screening molecules cannot be completely excluded. This potential limitation was considered when interpreting the prioritization results.

### 4.3. Drug Screening Guided by Fivefold Maximum Drug-Likeness

To implement screening guided by 5F-MDL, we developed a QSAR-based computational workflow using an ensemble of deep neural network predictors. The workflow consisted of three main modules: (i) construction and validation of endpoint-specific deep learning models for 33 molecular properties; (ii) prediction of a 33-dimensional property spectrum for each candidate molecule and each reference drug; and (iii) calculation of a final integrated similarity score, $S_{5F}$, based on the distance between candidate and reference profiles in the normalized property space. The overall objective was to rank candidate molecules according to their multidimensional similarity to clinically approved reference antibiotics across the five developability-related dimensions. The molecular representation, model construction, feature processing, scoring strategy, and robustness-control procedures are described below.

#### 4.3.1. Molecular Input and Representation

Each molecule was represented using 3599 two-dimensional molecular descriptors calculated with Dragon 7. These descriptors covered atom-level, bond-level, topological, constitutional, electronic, and whole-molecule features, and were used as input variables for endpoint-specific QSAR modeling. The same descriptor calculation procedure was applied to the reference drugs, training molecules, and candidate molecules to maintain consistency across model development and virtual screening. The descriptor-derived QSAR models were constructed for 33 molecular property endpoints spanning five developability-related dimensions: physicochemical properties, pharmacokinetics, efficacy-related properties, safety, and stability. The composition of these 33 property datasets and the corresponding predictive submodels is summarized in Table S1. Together, the predicted endpoints formed a 33-dimensional property spectrum intended to describe each molecule from multiple developability-related perspectives. This spectrum was then used for similarity-based prioritization under the 5F-MDL framework. It should be noted that this 33-dimensional spectrum represents the operational feature space used in the present implementation of 5F-MDL. It was selected according to endpoint relevance and the availability of QSAR-compatible datasets, and it was not intended to represent the only possible or optimal descriptor set for antibacterial prioritization.

#### 4.3.2. Supervised Training of the Deep Ensemble

Independent predictor submodels were trained for each of the 33 molecular property endpoints listed in Table S1. Training data were compiled from publicly available labeled molecular datasets, including ChEMBL, PubChem, ToxCast, the United States Environmental Protection Agency Distributed Structure-Searchable Toxicity (EPA DSSTox) database, and endpoint-specific QSAR literature sources. For each endpoint, the corresponding dataset was standardized and partitioned into training, validation, and held-out test subsets in an approximate ratio of 70:15:15. The downstream commercial screening libraries, including eMolecules, TargetMol, and MedChemExpress, were not intentionally incorporated as complete training sources during model construction. However, because both the endpoint datasets and the screening libraries cover broad public and commercial chemical space, limited molecule-level overlap or close structural relatedness could not be completely excluded. Therefore, the reported test-set performance should be interpreted as an internal held-out evaluation rather than as a strict external or scaffold-split validation. Feature selection was performed separately for each endpoint using the feature_selection module in scikit-learn under Python 3.6. Random forest models were first fitted to the descriptor matrix to estimate descriptor importance. The SelectFromModel procedure was then applied with the threshold set to “median”, retaining descriptors whose importance values met the median-based selection criterion estimated by the corresponding random forest model. This endpoint-wise feature-selection step was applied before deep learning model training to reduce feature redundancy and improve computational efficiency. The selected descriptor subset was therefore allowed to differ among endpoints, reflecting the fact that different molecular properties may depend on different structural features.

All 33 submodels adopted the same feedforward deep neural network architecture (Figure S1). The network contained three fully connected hidden layers with 2048, 1024, and 128 units, respectively. The rectified linear unit (ReLU) activation function was used in each hidden layer. Dropout with a rate of 0.2 and weight decay of $1\times 10^{-2}$ were applied to reduce overfitting. The third hidden layer, consisting of 128 units, served as a bottleneck representation to generate a compact latent feature representation before the output layer. For regression tasks, the output layer consisted of a single linear node. For classification tasks, the output layer consisted of a single node with sigmoid activation to estimate the probability of the positive class. Models were trained using the AdamW optimizer with a learning rate of $1\times 10^{-4}$, a batch size of 128, and early stopping with a patience of 10 epochs based on validation-set performance.

For regression tasks, the mean squared error (MSE) loss was used:

(1) $$MSE=\frac{1}{N_{batch}}\sum_{i=1}^{N_{batch}}{\left(y_{i}-{\hat{y}}_{i}\right)}^{2}$$

where $N_{batch}$ denotes the batch size, $y_{i}$ denotes the reference value, and ${\hat{y}}_{i}$ denotes the predicted value.

For classification tasks, the binary cross-entropy loss was used:

(2) $$L=-\frac{1}{N_{batch}}\sum_{i=1}^{N_{batch}}\left[y_{i}\log\left({\hat{y}}_{i}\right)+\left(1-y_{i}\right)\log\left(1-{\hat{y}}_{i}\right)\right]$$

where $y_{i}\in \{0,1\}$ denotes the reference label and ${\hat{y}}_{i}\in \left[0,1\right]$ denotes the predicted probability.

Model performance was evaluated on the held-out test subset for each endpoint. The coefficient of determination ($R^{2}$) was used for regression tasks, and the area under the receiver operating characteristic curve (AUC) was used for classification tasks. The predictive performance of the 33 endpoint-specific submodels is summarized in Table 1. These metrics were used to assess the internal predictive reliability of the submodels within the present workflow, while the need for stricter scaffold-aware and external validation is addressed in the Discussion. After training, the 33 submodels were used to generate a five-dimension-associated property spectrum for each molecule. For any molecule, the predicted spectrum was represented as a vector $V\in R^{33}$. Because the raw predicted properties span heterogeneous physical scales, direct comparison of unnormalized outputs would be dominated by large-magnitude quantities. Therefore, normalization was applied before similarity scoring. For regression-type properties spanning broad numerical ranges, such as minimum inhibitory concentration (MIC), inhibition constant ($K_{i}$), and median lethal dose (LD50), log transformation was applied during model training when appropriate. At prediction time, regression outputs were min–max normalized using the corresponding training-set label statistics and clipped to the unit interval:

(3) $$v_{k}=\mathrm{clip}\left(\frac{y_{k}-\min\left(Y_{train}^{k}\right)}{\max\left(Y_{train}^{k}\right)-\min\left(Y_{train}^{k}\right)},\;0,\;1\right)$$

where $y_{k}$ is the raw prediction for property k, $Y_{train}^{k}$ is the set of training labels for property *k*, and $\mathrm{clip}\left(x,0,1\right)=\max\left(0,\min\left(x,1\right)\right)$. This ensured that each normalized component $v_{k}$ was mapped to the range [0, 1]. For classification properties, such as human ether-à-go-go-related gene (hERG) risk and chemical stability, the predicted positive-class probability was used directly because it naturally lies in [0, 1].

Through these steps, each molecular property spectrum V was mapped to the 33-dimensional unit hypercube ${\left[0,1\right]}^{33}$. Because the values in V retain magnitude information for each predicted endpoint, a Euclidean distance-based measure was used to compute the final integrated similarity score, $S_{5F}$:

(4) $$S_{5F}\left(V_{cand},V_{ref}\right)=1-\frac{{\left\|V_{cand}-V_{ref}\right\|}_{2}}{\sqrt{N_{prop}}}$$

where $V_{cand}$ and $V_{ref}$ are the normalized property vectors of the candidate and reference molecules, respectively; $N_{prop}=33$ is the dimensionality of the property spectrum; and ${\left\|\cdot \right\|}_{2}$ denotes the Euclidean norm. The denominator $\sqrt{N_{prop}}$ corresponds to the maximum possible Euclidean distance in the 33-dimensional unit hypercube, ensuring that $S_{5F}$ lies within $\left[0,1\right]$. A score approaching 1 indicates high predicted similarity between the candidate and reference molecules across the 33 normalized endpoints. For each candidate molecule, the final prioritization score was defined as the maximum $S_{5F}$ value across the reference-drug profiles. The $S_{5F}$ score was used only as a multidimensional prioritization metric. It was not intended to represent direct antibacterial potency, binding affinity, clinical efficacy, or mechanistic validation. Therefore, molecules with high $S_{5F}$ values were considered suitable for downstream experimental evaluation, but high similarity scores alone were not interpreted as evidence of biological activity.

### 4.4. Candidate Prioritization and Prospective Evaluation

Based on the integrated similarity score $S_{5F}$, a ranking-based prioritization strategy was implemented. Candidate molecules were independently compared with each of the three reference-drug profiles in the normalized 33-dimensional property space. Within each reference-centered group, molecules were ranked in descending order of $S_{5F}$, and the top five candidates were retained. This procedure yielded 15 prioritized candidate molecules for subsequent prospective evaluation (Table 2). This reference-stratified selection was designed to retain molecules with high predicted multidimensional similarity to each reference antibiotic profile while avoiding selection from only a single reference-centered region of the property space. The procedure was therefore intended to support balanced reference-guided prioritization rather than to prove structural novelty or scaffold diversity. The prioritized candidates were evaluated using a staged workflow. First, all 15 candidates were assessed for in vitro antibacterial activity. Second, molecular docking was performed for the 15 candidates and the three reference antibiotics to examine their predicted noncovalent binding poses in the modeled PBP2 pocket. Third, molecular dynamics simulations and MM-PBSA analyses were performed for the M2–PBP2 complex and the cefuroxime–PBP2 reference complex to examine representative complex stability and interaction energetics. In addition, a Bocillin-FL competition assay was used to provide target-related biochemical support for the interaction between M2 and PBP2. These analyses were intended to provide prospective experimental and computational support for candidate prioritization, but they were not considered definitive external validation of the full 5F-MDL model or conclusive proof of antibacterial mechanism.

#### 4.4.1. Antibacterial Activity Testing

An adapted Kirby–Bauer disk diffusion assay was employed for preliminary antibacterial screening of the 15 prioritized candidates against *E. coli* ATCC 25922. The bacterial strain was inoculated into Mueller–Hinton broth (MHB), cultured at 37 °C to the logarithmic phase, and adjusted with sterile normal saline to a turbidity equivalent to a 0.5 McFarland standard corresponding to approximately $1.5\times 10^{8}$ colony-forming units per milliliter (CFU/mL). The adjusted bacterial suspension was then uniformly spread onto Mueller–Hinton agar (MHA) plates.

Candidate molecules and reference antibiotics were dissolved in dimethyl sulfoxide (DMSO), and sterile disks were loaded with each compound at 30 µg per disk. The final DMSO content was maintained below 1% and kept consistent across treatment groups to minimize solvent-related effects. The disks were placed on the inoculated MHA plates, which were then incubated in an inverted position at 37 °C for 16–18 h. After incubation, inhibition zone diameters were measured in millimeters.

All disk diffusion experiments were performed in triplicate as independent biological replicates. The inhibition zone diameters were reported as mean ± standard deviation (SD). Statistical significance among treatments was analyzed by one-way analysis of variance (ANOVA) followed by Tukey’s multiple-comparison test, with *p* < 0.05 considered statistically significant. Compounds exhibiting visible antibacterial activity were advanced to subsequent determination of the minimum inhibitory concentration (MIC) and minimum bactericidal concentration (MBC).

#### 4.4.2. Determination of Minimum Inhibitory Concentration and Minimum Bactericidal Concentration

On the basis of the disk diffusion results, candidates with visible antibacterial activity were further evaluated by broth microdilution in accordance with the Clinical and Laboratory Standards Institute (CLSI) M07-A9 guideline to determine the minimum inhibitory concentration (MIC). Stock solutions were prepared in dimethyl sulfoxide (DMSO) and serially twofold diluted in cation-adjusted Mueller–Hinton broth (CA-MHB) to yield final well concentrations ranging from 1.6 to 102.4 µg/mL. A bacterial inoculum adjusted to approximately $5\times 10^{5}$ colony-forming units per milliliter (CFU/mL) was added to compound-containing microplates, which were then incubated at 37 °C for 24 h. The MIC was defined as the lowest compound concentration at which no visible bacterial growth was observed by the unaided eye.

Following MIC determination, 10 µL from each well without visible growth was plated onto Mueller–Hinton agar and incubated at 37 °C for 24 h. The minimum bactericidal concentration (MBC) was defined as the lowest compound concentration resulting in a reduction in viable colony count of at least 99.9% compared with the initial inoculum. DMSO solvent controls and growth controls were included in each experiment. All MIC and MBC measurements were performed in three independent biological replicates.

#### 4.4.3. Molecular Docking Analysis

Molecular docking was performed as a supportive structure-based analysis to examine the potential noncovalent binding poses of the prioritized candidates and reference antibiotics in the modeled PBP2 pocket. The crystal structure of *E. coli* PBP2 was obtained from the Research Collaboratory for Structural Bioinformatics Protein Data Bank (RCSB PDB; PDB ID: 6G9P) in accordance with published procedures [69]. Before docking, the co-crystallized ligand and crystallographic water molecules were removed from the receptor structure. Three-dimensional structures of the 15 prioritized candidate molecules and the three reference antibiotics were prepared using ChemDraw Professional 15.0 and saved in Structure Data File (SDF) format. The same ligand-preparation procedure was applied to all candidate and reference molecules to maintain consistency across docking calculations.

Blind docking was conducted using the CB-Dock2 platform [70]. The platform identified five potential binding cavities in the receptor, with estimated volumes ranging from 286 to 635 Å^3^. Docking was then performed for each ligand, and the resulting docking scores and cavity assignments were recorded. The full docking score summary for the reference drugs and 15 candidate molecules is provided in Table S2. Docking output files were downloaded and inspected, and representative protein–ligand complexes and interaction patterns were visualized using PyMOL 3.1.0 and BIOVIA Discovery Studio 2021. The docking analysis was used to generate structural hypotheses regarding potential binding orientations and noncovalent interactions. Docking scores and predicted poses were not interpreted as definitive evidence of binding affinity, target inhibition, or antibacterial mechanism.

#### 4.4.4. Molecular Dynamics Simulation and MM-PBSA Analysis

Molecular dynamics simulations were performed to examine the conformational stability and interaction patterns of representative protein–ligand complexes under explicit-solvent simulation conditions. The M2–PBP2 complex, representing the most active candidate in the present experimental evaluation, and the cefuroxime–PBP2 reference complex were selected for simulation. The simulations were carried out using the AMBER 22 software suite. Protein parameters were assigned using the ff14SB force field. Ligand parameters were generated using the General AMBER Force Field 2 (GAFF2). Restrained electrostatic potential (RESP) charges for the ligands were derived after quantum-chemical calculations in Gaussian 16 at the HF/6-31G* level of theory. Each system was solvated in a TIP3P water box with a 10 Å buffer distance, and sufficient ${\mathrm{Na}}^{+}$ and ${\mathrm{Cl}}^{-}$ ions were added to neutralize the total system charge.

To remove unfavorable contacts and achieve equilibration, a standard multistep protocol was applied. First, two-stage energy minimization was performed, consisting of restrained minimization with positional restraints on the solute to relax the solvent, followed by unrestrained minimization of the whole system. The systems were then heated from 0 to 300 K over 50 ps under the constant-volume ensemble (NVT), followed by 200 ps of equilibration under the constant-pressure ensemble (NPT) at 1.0 atm and 300 K. Production simulations were subsequently performed for 100 ns under NPT conditions with all restraints removed. A 2 fs time step was used, corresponding to $5\times 10^{7}$ steps for each production run, and trajectories and energies were saved every 5000 steps. To improve statistical robustness, three independent simulations were performed for each complex using different random seeds.

The simulations were designed to evaluate the stability of representative bound complexes after ligand placement in the modeled PBP2 pocket. They were not intended to reproduce the ligand concentrations used in the Bocillin-FL competition assay or to model concentration-dependent competition in bulk solution. Such concentration-dependent behavior would require a different computational design, such as multi-ligand competitive simulations or free-energy/kinetic modeling, which was beyond the scope of the present study. Simulation trajectories were analyzed using the cpptraj module. Backbone root-mean-square deviation (RMSD) and per-residue root-mean-square fluctuation (RMSF) were calculated to assess overall complex stability and residue-level flexibility. Protein–ligand noncovalent interactions were further analyzed using the final 10 ns of each production trajectory, corresponding to 1000 frames per trajectory, to obtain interaction occupancies for the equilibrated simulation segment. Hydrogen bonds were identified using a donor–acceptor distance cutoff of 3.5 Å and a donor–hydrogen–acceptor angle cutoff of 120°. Hydrophobic contacts were recorded using a heavy-atom distance cutoff of 4.5 Å between nonpolar protein residues or side-chain atoms and hydrophobic regions of the ligand. π–π interactions were identified using ring-centroid distance and ring-orientation criteria consistent with stacked or T-shaped π–π interactions. Interaction occupancy was calculated as the percentage of analyzed trajectory frames in which a given interaction was present. The mean number of hydrogen bonds and the occupancies of major hydrogen-bonding, hydrophobic, and π–π interactions are summarized in Table 5. Binding free energies were estimated using the molecular mechanics Poisson–Boltzmann surface area (MM-PBSA) method. For each trajectory, the final 10 ns of the production simulation was used for MM-PBSA analysis, corresponding to 1000 frames. The total binding free energy ($\Delta G_{bind}$) and its major energy components, including van der Waals, electrostatic, polar solvation, and nonpolar solvation contributions, were calculated. In this study, the polar and nonpolar solvation terms were used to describe solvation-related contributions associated with desolvation effects. The MM-PBSA energy components are summarized in Table 6. The molecular dynamics and MM-PBSA analyses were used as supportive computational assessments of complex stability and interaction energetics. These analyses were not interpreted as definitive evidence of PBP2 inhibition or as conclusive validation of the antibacterial mechanism of M2.

#### 4.4.5. Bocillin-FL-Based PBP2 Competition Labeling Assay

Expression and purification of recombinant *E. coli* PBP2 were carried out with reference to the methods reported by Levy, Nygaard, and co-workers, with appropriate modifications made for the present study [71,72]. A Bocillin-FL-based competition labeling assay was used to examine whether the candidate compound M2 could interfere with fluorescent probe labeling of PBP2 at or near the active-site region. This assay was used as target-related biochemical support and was not intended to provide a direct measurement of binding affinity or functional enzyme inhibition.

Purified PBP2 was added to the reaction mixture at a final concentration of 50 µg/mL and was first pre-incubated with M2 at final concentrations of 25, 50, and 100 µM in reaction buffer at 37 °C for 10 min. Bocillin-FL was then added to a final concentration of 5 µM, followed by further incubation at 37 °C for 10 min to allow the labeling reaction to proceed. Cefuroxime (50 µM) was used as the positive control, and DMSO was used as the solvent control, with the final DMSO concentration maintained below 1%. The total reaction volume was 20 µL.

Reactions were terminated by adding 5× SDS loading buffer and heating at 95 °C for 5 min. Samples were separated by 4–12% SDS–polyacrylamide gel electrophoresis (SDS–PAGE). Bocillin-FL fluorescence signals were acquired before protein staining using a Tanon 5200 multifunctional gel imaging system (Shanghai Tianneng Technology Co., Ltd., Shanghai, China). The same gel was then stained with Coomassie Brilliant Blue to verify consistency of total protein loading. Fluorescence band intensities were quantified using ImageJ software (version 1.53t; National Institutes of Health, Bethesda, MD, USA), normalized to the corresponding total protein band intensities, and expressed relative to the DMSO control group.

All assays were performed in three independent experiments. Data are presented as mean ± standard deviation (SD). Statistical differences among groups were analyzed using one-way analysis of variance (ANOVA) followed by Dunnett’s multiple-comparison test versus the DMSO control, with *p* < 0.05 considered statistically significant.

## 5. Conclusions

In this study, we developed a 5F-MDL workflow for early-stage antibacterial candidate prioritization against *Escherichia coli*. By integrating 33 predicted molecular properties across five developability-related dimensions, this workflow enabled the prioritization of candidate molecules from a large commercial screening space and provided preliminary proof-of-concept support for multidimensional, developability-aware screening. Among the 15 prioritized candidates, M2 showed the most favorable overall in vitro profile under the tested conditions, with measurable inhibitory and bactericidal activity against *E. coli* ATCC 25922. However, its potency remained substantially weaker than that of cefuroxime, and it should therefore be regarded as a preliminary antibacterial hit for further optimization rather than as a fully validated lead compound. Docking, molecular dynamics, MM-PBSA, and Bocillin-FL competition analyses provided target-related supportive evidence for interaction with the modeled PBP2 active-site region, but they did not conclusively establish PBP2 as the primary intracellular target or define the precise mechanism of action. Overall, 5F-MDL may serve as an exploratory framework for multidimensional antibacterial candidate prioritization. Its broader applicability, comparative advantage, and mechanistic relevance require further validation using larger prospective compound sets, resistant clinical isolates, systematic benchmarking, and direct target-validation experiments.

## Figures and Tables

**Table 1:** Predictive performance of the 33 property-specific deep learning submodels on independent test sets, grouped by the five developability-related dimensions of the 5F-MDL framework.

Domain	Submodel	Endpoint	Task Type	$\mathrm{Performance}\;(R^{2}$/AUC)
Physicochemical properties	S1	Aqueous solubility (log S)	Regression	0.87
	S2	Octanol–water partition coefficient (log P)	Regression	0.89
	S3	Melting point	Regression	0.86
	S4	Boiling point	Regression	0.92
	S5	Surface tension	Regression	0.94
	S6	Density	Regression	0.89
	S7	Viscosity	Regression	0.88
	S8	Flash point	Regression	0.91
	S9	Vapor pressure	Regression	0.93
	S10	Dissociation constant	Regression	0.95
	S11	Hydrolysis (half-life value)	Regression	0.92
Pharmacokinetics	S12	Bioavailability	Regression	0.88
	S13	Plasma protein binding rate	Regression	0.86
	S14	Maximal rate of metabolism	Regression	0.87
Pharmacokinetics	S15	Biliary excretion rate	Regression	0.86
	S16	Urinary excretion rate	Regression	0.88
	S17	Volume of distribution	Regression	0.89
	S18	Half-life	Regression	0.89
Efficacy	S19	Minimum Inhibitory Concentration (MIC)	Regression	0.93
	S20	Enzyme inhibition constant (Ki)	Regression	0.95
	S21	Receptor affinity	Regression	0.91
	S22	Maximum effect model parameter (Emax)	Regression	0.93
	S23	50% effective dose (EC50)	Regression	0.92
Safety	S24	Median lethal dose (LD50)	Regression	0.93
	S25	No Observed Adverse Effect Level (NOAEL)	Regression	0.91
	S26	Tetrahymena pyriformis 50% growth inhibition concentration	Regression	0.92
	S27	Median lethal concentration (LC50)	Regression	0.96
	S28	Developmental toxicity	Classification	0.93
	S29	Ames mutagenicity	Classification	0.96
	S30	hERG risk	Classification	0.95
Stability	S31	Chemical stability	Classification	0.96
	S32	Thermostability	Classification	0.91
	S33	Light stability	Classification	0.92

Notes: Overall, the 27 regression submodels showed $R^{2}$ values ranging from 0.86 to 0.96, whereas the 6 classification submodels showed AUC values ranging from 0.91 to 0.96. $R^{2}$, coefficient of determination; AUC, area under the receiver operating characteristic curve.

**Table 2:** Overview of the top fifteen candidate molecules prioritized by the 5F-MDL strategy.

Compound	Smiles	$S_{5F}$	Nearest Drug	Euclidean Distance
M1	Cc1cc(C)n(n1)S(=O)(=O)c2ccc3c4ccc(cc4(C(=O)c3(c2)))S(=O)(=O)n5nc(C)cc5(C)	0.956	Ceftriaxone	0.252
M2	COc1ccc(cc1S(=O)(=O)NCc2ccc(cc2)S(N)(=O)=O)N3C(=O)CCS3(=O)(=O)	0.956	Cefuroxime	0.253
M3	CC7=C(c5csc(NC(=O)c4c(N)c(C(=O)c2nc1ccccc1s2)sc4(Nc3ccccc3))n5)C(=O)N(c6ccccc6)N7(C)	0.956	Cefradine	0.254
M4	O=C(CN2C(=O)C(=Cc1ccccc1)SC2=S)NN5C(=O)CS(=C4C(=O)Nc3ccccc34)C5=S	0.952	Cefradine	0.274
M5	OC(=O)c1ccc(cc1)C(=O)c3c5c(cc2C(=O)N(C(=O)c23)c4nccs4)C(=O)N(C5(=O))c6nccs6	0.951	Cefuroxime	0.281
M6	CCOc1ccc3c(c1)C4=C(C(C)(C)N3(C(=O)c2ccccc2))SC(C(=O)OC)=C(C(=O)OC)C45(SC(C(=O)OC)=C(C(=O)OC)S5)	0.950	Cefradine	0.286
M7	O=C(Nc1nccs1)c2ccc3c(c2)C(=O)N(C3(=O))c4cccc(n4)N6C(=O)c5ccc(cc5C6(=O))C(=O)Nc7nccs7	0.950	Cefuroxime	0.287
M8	Cc1cc(C)nc(n1)NS(=O)(=O)c2ccc(cc2)NC=C(C#N)c3nc(cs3)C4=Cc5cc(ccc5(OC4(=O)))[N+](=O)[O-]	0.950	Cefradine	0.290
M9	C=CCN5C(=O)C(=C2N(C)c1ccccc1S2)SC5(=C4C(C)=NN(c3cccc(c3)S(O)(=O)=O)C4(=O))	0.949	Cefuroxime	0.292
M10	COC(=O)C4=C(C(=O)OC)SC3=C(c1ccccc1N(C(=O)c2cccs2)C3(C)(C))C45(SC(C(=O)OC)=C(C(=O)OC)S5)	0.946	Cefradine	0.313
M11	O=C(CCN3C(=O)C(=Cc1ccc2OCOc2(c1))SC3=S)Nc4cc(ccc4(O))[N+](=O)[O-]	0.945	Cefuroxime	0.318
M12	O=C(CN2C(=O)c1ccccc1S2(=O)(=O))NN4C(=O)C(=Cc3ccc(cc3)[N+](=O)[O-])SC4=S	0.944	Ceftriaxone	0.320
M13	[H]C2(NC(=O)Cc1ccccc1)(C(=O)N(NC(=O)OCC[Si](C)(C)C)C2C4([H])(ON=CC(OC(=O)c3ccccc3)C4(C)(O)))	0.942	Ceftriaxone	0.334
M14	NS(=O)(=O)c1ccc(cc1)NC(=O)CSc3nc(SCC(=O)Nc2ccc(cc2)S(N)(=O)=O)sn3	0.941	Ceftriaxone	0.337
M15	CC(=O)OCC2OC(NC1=C(C(=O)N(C)C(=O)N1(C))[N+](=O)[O-])C(OC(C)=O)C(OC(C)=O)C2(OC(C)=O)	0.936	Ceftriaxone	0.370

Notes: The table summarizes the top fifteen candidate molecules prioritized by the 5F-MDL strategy. The similarity score ($S_{5F}$) reflects the global similarity between each candidate and its nearest reference drug in the normalized 33-dimensional predicted property space. SMILES strings are provided to define the molecular structures of the prioritized candidates.

**Table 3:** MICs and MBCs of prioritized candidate molecules against *Escherichia coli* ATCC 25922.

Compound	MIC (µg/mL)	MBC (µg/mL)
M2	25.6	51.2
M8	25.6	102.4
M9	51.2	102.4
Cefuroxime	3.2	6.4

Notes: The antibacterial activities of the candidate molecules are compared with those of the reference antibiotics. MIC, minimum inhibitory concentration; MBC, minimum bactericidal concentration.

**Table 4:** Summary of docking results for the three phenotypically active candidate molecules.

Compound	Nearest Drug	Best Docking Score (kcal/mol)
M2	Cefuroxime	−9.8
M8	Cefradine	−11.3
M9	Cefuroxime	−10.5

Notes: The table summarizes the docking outcomes of the three phenotypically active candidate molecules selected for further analysis. Although M8 showed the most favorable docking score, M2 displayed the most balanced overall biological profile under the tested conditions.

**Table 5:** Quantitative interaction summary of the cefuroxime–PBP2 and M2–PBP2 complexes from molecular dynamics simulations.

Complex	Mean H-Bond No.	H-Bond (s)	Hydrophob.	π–π
Cefuroxime-PBP2	1.773	PRO66 (78.0%) ARG68 (99.3%)	ALA65 (97.2%) TYR161 (90.5%) ALA201 (84.2%)	TYR161 (3.6%)
M2-PBP2	2.634	PRO63 (66.9%) LYS162 (97.9%) THR202 (98.6%)	ILE64 (3.9%) ALA65 (99.1%) TYR161 (82.4%) LYS162 (1.3%) ALA201 (7.5%)	TYR161 (12.7%)

Note: The percentages in parentheses represent interaction occupancies over the analyzed molecular dynamics trajectories. All observed interactions from the present analysis are listed, including low-occupancy contacts. Mean H-Bond No., mean number of conventional hydrogen bonds; H-Bond(s), conventional hydrogen-bonding interactions; Hydrophob., hydrophobic contacts; π–π, π–π stacking interactions.

**Table 6:** MM-PBSA energy components of the cefuroxime–PBP2 and M2–PBP2 complexes.

Complex	ΔG_bind	vdW	Elec	Pol. solv.	Npol. solv.
Cefuroxime-PBP2	−29.40	−35.40	−18.22	+27.08	−2.86
M2-PBP2	−38.54	−45.21	−28.65	+39.12	−3.80

Note: All energy terms are given in kcal/mol. ΔG_bind, binding free energy; vdW, van der Waals contribution; Elec, electrostatic contribution; Pol. solv., polar solvation contribution; Npol. solv., nonpolar solvation contribution.

## Data Availability

The original contributions presented in this study are included in the article/Supplementary Materials. Further inquiries can be directed to the corresponding authors.

## Supplementary Materials

The following supporting information can be downloaded at: https://www.mdpi.com/article/10.3390/ph19050744/s1, Figure S1: Canonical architecture of an individual deep learning submodel used for property prediction; Figure S2: Bocillin-FL competition assay evaluating the interaction of M2 with *E. coli* PBP2; Figure S3: Full uncropped source images corresponding to Figure S2A; Table S1: Composition of the 33 property datasets and overview of the corresponding predictive submodels; Table S2: Molecular docking scores of the reference drugs and the top fifteen candidate molecules; Table S3: Selected SwissADME-predicted developability-related descriptors for cefuroxime, M2, M8, and M9.
