# Supplementary material for: A Fivefold Maximum Drug-Likeness Strategy for Prioritizing Antibacterial Candidates Against Escherichia coli
Source: Pharmaceuticals (Basel). 2026 May 8;19(5):744. doi: 10.3390/ph19050744 (PMC13209408; doi:10.3390/ph19050744)
Supplement: Supplementary file 1 [file pharmaceuticals-19-00744-s001.zip › pharmaceuticals-4257352-supplementary.pdf]

Supporting Information

A Fivefold Maximum Drug-Likeness Strategy for Prioritizing Antibacterial Candidates Against Escherichia coli

Haoyu Zhu[1], Shijie Du[1], Qin Yang[2], Lu Xu[1,3], Wei Shi[1]

[1] College of Material and Chemical Engineering, Tongren University, Tongren 554300, PR China

[2] School of Physics and Optoelectronic Engineering, Yangtze University, Jingzhou 434023, PR China

[3] School of Sports and Health Science, Tongren University, Tongren 554300, PR China

Correspondence: chyxl@gztrc.edu.cn (Xu L.); chyshw@gztrc.edu.cn (Shi W.)

CONTENTS.

Figure S1. Canonical architecture of an individual deep learning submodel used for property prediction.

Figure S2. Bocillin-FL competition assay evaluating the interaction of M2 with *Escherichia coli* PBP2.

Table S1. Composition of the 33 property datasets and overview of the corresponding predictive submodels.

Table S2. Molecular docking scores of the reference drugs and the top fifteen candidate molecules.

Table S3. Selected SwissADME-predicted developability-related descriptors for cefuroxime, M2, M8, and M9.

Figure S3. Full uncropped source images corresponding to Figure S2A

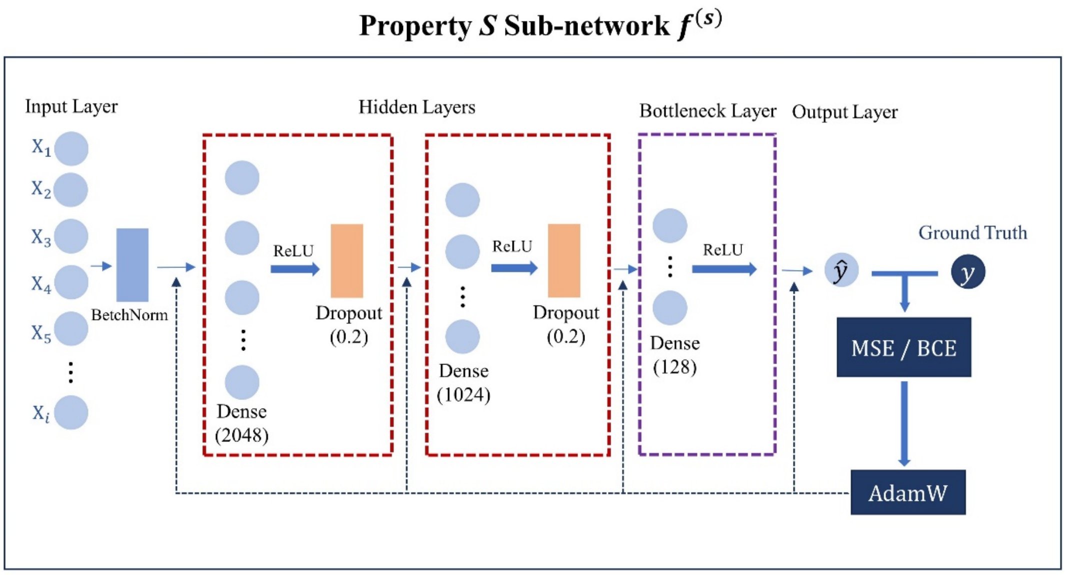

Figure S1. Canonical architecture of an individual deep learning submodel used for property prediction. Each submodel consists of three fully connected hidden layers with 2,048, 1,024, and 128 units, respectively, employing ReLU activation functions. The network is trained independently for each property to enable modular construction of the 33-dimensional drug-likeness spectrum.

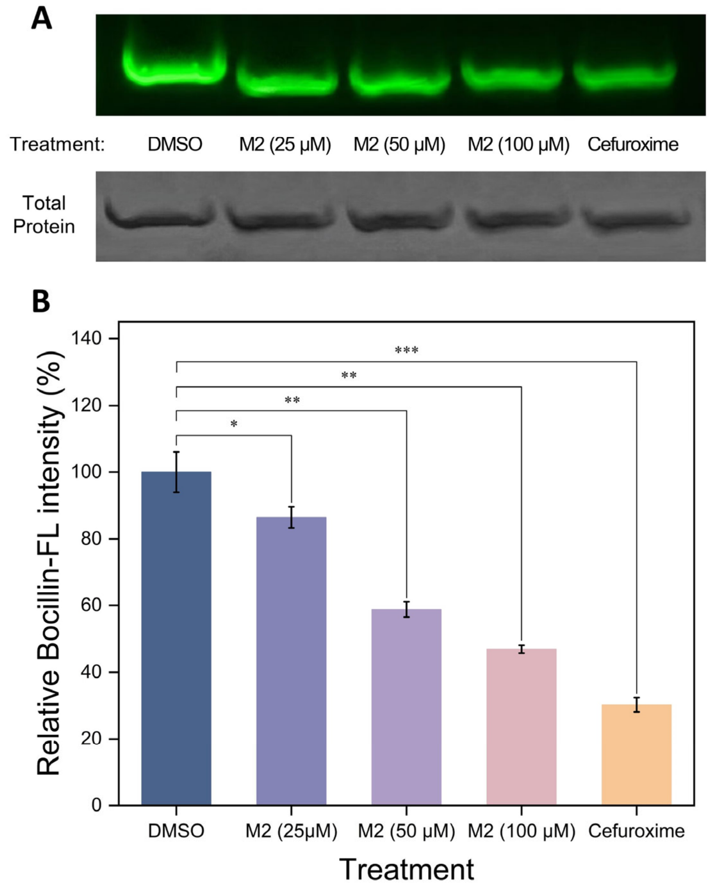

Figure S2. Bocillin-FL competition assay evaluating the interaction of M2 with Escherichia coli PBP2. (A) Representative fluorescent gel image showing Bocillin-FL labeling of purified PBP2 after treatment with DMSO, M2 (25, 50, and 100 μM), or cefuroxime. The lower panel shows Coomassie Brilliant Blue staining of the same gel to confirm comparable protein loading. (B) Quantitative analysis of relative Bocillin-FL intensity. Fluorescence intensities were normalized to the corresponding total protein band intensity and expressed relative to the DMSO control group. Data are presented as mean ± SD from three independent experiments. Statistical significance was determined by one-way ANOVA followed by Dunnett's multiple comparison test versus the DMSO control (* $p < 0.05$, ** $p < 0.01$, *** $p < 0.001$).

Table S1. Composition of the 33 property datasets and overview of the corresponding predictive submodels.

No.	Name	Class	Training set	Validation set	Testing set
S1	Aqueous solubility (LogS)	C1	251267	53847	53851
S2	Octanol–water partition coefficient (log P)	C1	286544	61254	61263
S3	Melting point	C1	130559	27972	27984
S4	Boiling point	C1	125784	26958	26962
S5	Surface tension	C1	59168	12679	12697
S6	Density	C1	167325	35846	35869
S7	Viscosity	C1	109081	23374	23392
S8	Flash point	C1	161846	34675	34683
S9	Vapor pressure	C1	137928	29558	29564
S10	Dissociation constant	C1	257753	55163	55319
S11	Hydrolysis (half-life value)	C1	117046	25098	25183
S12	Bioavailability	C2	39298	8417	8461
S13	Plasma protein binding rate	C2	77264	16547	16583
S14	Maximal rate of metabolism	C2	102937	22054	22068
S15	Biliary excretion rate	C2	44518	9539	9671
S16	Urinary excretion rate	C2	57047	12248	12239
S17	Volume of distribution	C2	137489	29439	29586
S18	Half-life	C2	129632	27747	27864
S19	Minimum Inhibitory Concentration (MIC)	C3	335587	71956	71998
S20	Enzyme inhibition constant（Ki）	C3	137418	29447	29456
S21	Receptor affinity	C3	185813	39736	39973
S22	Maximum effect model parameter (Emax)	C3	48246	10348	10369
S23	50% effective dose（EC50）	C3	161724	34648	34623
S24	Median lethal dose (LD50)	C4	161591	34627	34654
S25	No Observed Adverse Effect Level (NOAEL)	C4	206658	44246	44289
S26	Tetrahymena pyriformis 50% growth inhibition	C4	245736	52639	52784

	concentration				
S27	Median lethal concentration (LC50)	C4	155369	33247	33286
S28	Developmental toxicity	C4	257561	55142	55364
S29	Ames mutagenicity	C4	397734	85237	85368
S30	hERG_risk	C4	174397	37296	37412
S31	Chemical stability	C5	249362	53447	53586
S32	Thermostability	C5	335341	71839	71964
S33	Light stability	C5	33789	7228	7243

Note: The table summarizes the classification of properties (where C1 denotes physicochemical properties, C2 pharmacokinetics, C3 efficacy, C4 safety, and C5 stability) and lists the corresponding training datasets and model configurations.

Table S2. Molecular docking scores of the reference drugs and the top fifteen candidate molecules.

Cavity Volumes									
517 Å^3		549 Å^3		286 Å^3		635 Å^3		510 Å^3	
Compd. no	kcal/mol	Compd. no	kcal/mol	Compd. no	kcal/mol	Compd. no	kcal/mol	Compd. no	kcal/mol
Cefradine	-8.2	Cefradine	-7.6	Cefradine	-7.3	Cefradine	-6.5	Cefradine	-6.1
Cefuroxime	-8.5	Cefuroxime	-7.6	Cefuroxime	-6.5	Cefuroxime	-6.1	Cefuroxime	-6.8
Ceftriaxone	-8.4	Ceftriaxone	-8.2	Ceftriaxone	-7.5	Ceftriaxone	-7.2	Ceftriaxone	-7.9
M1	-10.9	M1	-10.0	M1	-9.2	M1	-8.7	M1	-8.2
M2	-9.8	M2	-7.7	M2	-7.8	M2	-7.7	M2	-7.0
M3	-11.2	M3	-9.9	M3	-9.5	M3	-8.5	M3	-9.2
M4	-9.0	M4	-8.8	M4	-7.3	M4	-7.2	M4	-7.8
M5	-9.1	M5	-8.9	M5	-8.2	M5	-7.5	M5	-7.8
M6	-10.2	M6	-7.2	M6	-5.5	M6	-5.4	M6	-6.4
M7	-11.0	M7	-10.2	M7	-10.6	M7	-8.8	M7	-9.4
M8	-11.3	M8	-10.0	M8	-11.2	M8	-9.0	M8	-8.7
M9	-10.5	M9	-8.7	M9	-8.9	M9	-7.4	M9	-7.8
M10	-8.7	M10	-10.2	M10	-7.1	M10	-6.9	M10	-6.2
M11	-9.5	M11	-8.5	M11	-8.5	M11	-7.4	M11	-7.3
M12	-9.1	M12	-9.0	M12	-8.5	M12	-7.8	M12	-7.8
M13	-9.0	M13	-8.0	M13	-7.8	M13	-6.4	M13	-6.4
M14	-9.3	M14	-8.3	M14	-8.6	M14	-7.1	M14	-7.1
M15	-9.0	M15	-7.2	M15	-6.6	M15	-6.2	M15	-6.2

Note: The table lists the lowest predicted binding energies (kcal/mol) within the primary binding cavity of PBP2, providing quantitative support for the docking analysis discussed in the main text.

Table S3. Selected SwissADME-predicted developability-related descriptors for cefuroxime, M2, M8, and M9.

Compound	MW	H-Bond Acceptors/ Donors	TPSA	Consensus Log P	ESOL Class	GI absorption	Bioavailability Score	Brenk alerts	Lead-likeness violations	Synthetic Accessibility
Cefuroxime	424.39	9/3	199.06	-0.26	Very soluble	Low	0.11	2	2	4.74
M2	489.54	10/2	195.15	0.11	Soluble	Low	0.55	0	1	3.52
M8	601.61	10/2	233.31	3.04	Poorly soluble	Low	0.17	4	3	4.35
M9	540.63	6/1	178.83	3.04	Poorly soluble	Low	0.17	4	3	4.35

Note: The table summarizes selected SwissADME-predicted physicochemical and developability-related descriptors for cefuroxime and the three phenotypically active candidate molecules (M2, M8, and M9). These descriptors were included to facilitate comparison of solubility-related, polarity-related, and medicinal-chemistry-related features that may contribute to differences in phenotypic antibacterial performance. MW, molecular weight; H-bond Acceptors/Donors, number of hydrogen-bond acceptors and hydrogen-bond donors; TPSA, topological polar surface area; Consensus Log P, consensus octanol/water partition coefficient; ESOL Class, solubility class predicted by the ESOL model; GI absorption, predicted gastrointestinal absorption; Bioavailability Score, predicted oral bioavailability score; Brenk alerts, number of Brenk structural alerts; Lead-likeness violations, number of lead-likeness rule violations; Synthetic Accessibility, synthetic accessibility score.

Full uncropped source images for Figure S2A

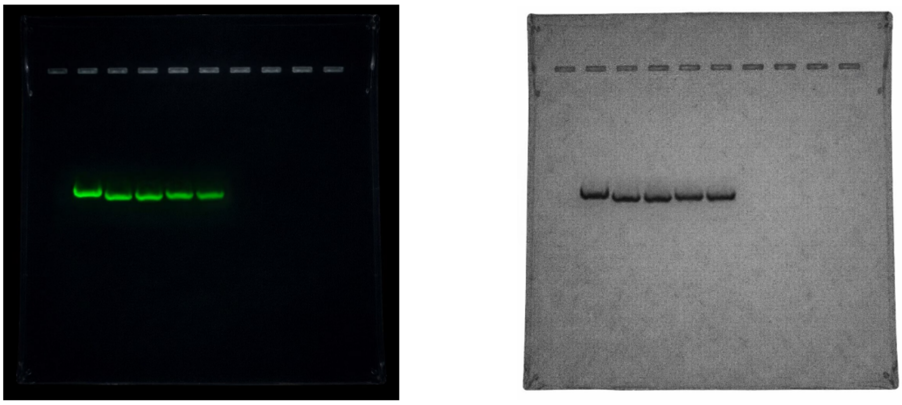

(a) (b)

Full uncropped source images corresponding to Figure S2A in the main manuscript are provided, where (a) shows the Bocillin-FL fluorescence image and (b) shows the Total Protein image obtained from the same gel. From left to right, the lanes correspond to the DMSO group, M2 (25 μM) group, M2 (50 μM) group, M2 (100 μM) group, and the cefuroxime group.

Figure S3. Full uncropped source images corresponding to Figure S2A. (a) shows the Bocillin-FL fluorescence image and (b) shows the Total Protein image obtained from the same gel. From left to right, the lanes correspond to the DMSO group, M2 (25 μM) group, M2 (50 μM) group, M2 (100 μM) group, and the cefuroxime group.
